# Pro-death and pro-survival properties of ouabain in U937 lymphoma derived cells

**DOI:** 10.1186/1756-9966-31-95

**Published:** 2012-11-15

**Authors:** Francesca Cuozzo, Marisa Raciti, Laura Bertelli, Rosanna Parente, Livia Di Renzo

**Affiliations:** 1 Department of Experimental Medicine, University of Rome “La Sapienza”, Viale Regina Elena 324, Rome 00161, Italy

**Keywords:** Ouabain, Ca^++^, NCX, p38 MAPK, Cell death, Cell survival, Lymphoma

## Abstract

**Background:**

Epidemiological studies revealed significantly lower mortality rates in cancer patients receiving cardiac glycosides, which turned on interest in the anticancer properties of these drugs. However, cardiac glycosides have also been shown to stimulate cell growth in several cell types. In the present investigation we analyzed the pro-death and pro-survival properties of ouabain in the human lymphoma derived cell line U937.

**Methods:**

ROS, intracellular Ca^++^, cell cycle were evaluated by loading the cells with fluorescent probes under cytofluorimetry. Cell counts and evaluation of trypan blue-excluding cells were performed under optic microscope. Protein detection was done by specific antibodies after protein separation from cellular lysates by SDS-PAGE and transfer blot.

**Results:**

High doses of ouabain cause ROS generation, elevation of [Ca^++^]_i_ and death of lymphoma derived U937 cells. Lower doses of OUA activate a survival pathway in which plays a role the Na^+^/Ca^++^-exchanger (NCX), active in the Ca^++^ influx mode rather than in the Ca^++^ efflux mode. Also p38 MAPK plays a pro-survival role. However, the activation of this MAPK does not appear to depend on NCX.

**Conclusion:**

This investigation shows that the cardiac glycoside OUA is cytotoxic also for the lymphoma derived cell line U937 and that can activate a survival pathway in which are involved NCX and p38 MAPK. These molecules can represent potential targets of combined therapy.

## Background

The Na^+^/K^+^ ATPase catalyzes the electrogenic exchange of three intracellular Na^+^ ions for two extracellular K^+^ ions using for this transport energy that is released from the hydrolysis of ATP. In this way Na^+^/K^+^ ATPase plays an important role in the regulation of intracellular Na^+^ and K^+^ concentrations and in the maintenance of electrical membrane potential, cell volume, and Na^+^-coupled transport of amino acids, glucose, nucleotides, and other compounds with low molecular mass [1-3].

Ouabain (OUA) is a cardiac glycoside that has been used for long time for the treatment of cardiac insufficiency. OUA by binding to the α-subunit of Na^+^/K^+^ ATPase inhibits it. The inhibition of the Na^+^/K^+^ ATPase, reducing the sodium gradient, leads to increased cytosolic [Ca^++^ probably by impairing the activity of the Na^+^/Ca^++^-exchanger (NCX) [4-9]. NCX is one of the main pathways for intracellular Ca^++^ clearance [9] and the inhibition of the Na^+^/K^+^ ATPase by cardiac glycosides, causing the inversion of the Na^+^/K^+^ gradient, leads to impairment of the NCX activity, contributing to accumulation of Ca^++^[4-9].

Results from epidemiological studies showed significantly lower mortality rates in cancer patients receiving cardiac glycosides, which turned on interest in the antineoplastic properties of these drugs [10]. In various cancer cell lines, including prostate cancer cells or breast tumor cells, cardiac glycosides induce apoptosis [11-16]. These glycosides are considered to be cytotoxic for tumors because malignant cells express high levels of Na^+^/K^+^ ATPase α-isoforms, which are inhibited by them [17]. However, cardiac glycosides induce complex signaling cascades that lead to a variety of effects including the induction of proliferation on vascular smooth muscle cells [18], lymphocytes [19], prostate cells [20] and HeLa cells [21]. It appears that cardiac glycosides affect multiple signaling pathways, suggesting that their anti-cancer effect may be multifactorial and context dependent. To clarify the pro-survival or pro-death properties of OUA in the lymphoma derived U937 cells, we set out to investigate how high doses and low doses of the drug affect these parameters. Interestingly, by this means we detected that high doses of OUA are cytotoxic also for U937 cells, while low doses of OUA cause a rise of cytoplasmic Ca^++^ through NCX which appears to counter cell death. We detected also the activation and the pro-survival role of p38 MAPK upon OUA treatment, which appears to be NCX independent.

## Methods

### Reagents

RPMI 1640, fetal calf serum, l-glutamine, penicillin-streptomycin, phosphate buffered saline (PBS), ouabain, monensin, tunicamycin and antibodies anti β-actin were from Sigma-Aldrich (St. Louis, MO, USA). Anisomycin, SB203580 and PD98059 were from Calbiochem (Inalco, Milan, Italy). KB-R7943 was from Tocris (Cookson Inc., Ellisville, MO, USA). Antibodies anti phospho-p38 and anti p38 were from Cell Signaling Technology (Beverly, MA). Horseradish peroxidase (HRP)-conjugated anti-immunoglobulin antibodies, enhanced chemiluminescence (ECL) reagents and Hyperfilm-ECL film were from Amersham (Arlington Heights, IL, USA). Protein standards for SDS-polyacrylamide gel electrophoresis (SDS-PAGE) and nitrocellulose membranes were from Bio-Rad (Segrate, Milan, Italy). The membrane permeant CDCF-DA and FLUO-3-AM were from Molecular Probes (SIC, Rome, Italy), and other reagents were of the highest purity and purchased from Bio-Rad or Sigma.

### Cell viability and growth

U937 cells, derived from the pleural effusion of a patient with histiocytic lymphoma [22], were grown in complete medium (RPMI-1640 medium supplemented with 1.0% sodium pyruvate, 5% FCS, 2 μM glutamine, 100 units/ml penicillin and 100 μg/ml streptomycin) at 37°C, in fully humidified atmosphere 95% room air/5% CO_2_. Cells were resuspended three times a week in fresh complete medium as 3×10^5^/ml. Cell growth was evaluated by hemocytometry counts of cells excluding Trypan blue (0.04% Trypan blue in PBS, w/v), and viability was assessed by calculating alive (trypan blue-excluding) cells as percentage of all cells counted. Cells used in every experiment were ≥93% viable and taken from cultures in exponential growth. They were washed once and resuspended in complete medium, 1×10^6^/ml, and transferred to 24-well microplates. They were then treated with inhibitors or vehicles, incubated for 30 min, and susequently exposed to test agents or, again, to vehicles. At the end of each experiment, the cells were gently mixed and aliquots were taken for cell counting and cell cycle analysis. The vehicles, even when used in combination, were ≤0.3% (v/v) and did not modify any investigated parameter in comparison with control culture.

### Flow cytometric analysis of cell death

Nuclear DNA fragmentation was quantified by flow cytometry of hypodiploic (subG1) DNA after cell fixation and staining with PI [23,24]. Briefly, cells were washed with PBS, pelletted and fixed in ice cold ethanol/water (70/30, v/v) for 1 h, pelletted again and washed twice with PBS, and finally resuspended in PBS containing RNAse (20 μg/ml) and PI (100 μg/ml). Events in the different cell cycle phases were gated manually using an EPICS XL cytofluorimeter (Beckman Coulter, Hialeah, Fl, USA). At least 10.000 events/sample were acquired. Collected data were analysed using the Multicycle software for DNA content and cell cycle analysis (Phoenix Flow System, San Diego, CA, USA). The subG1 events representative of the apoptotic cells, and the events in the other cell cycle phases, are given as a percentage of the total cell population.

### Western blot analysis

Whole cell lysates were prepared as previously described [25,26]. Briefly, the cells were kept for 30 min on ice in lysis buffer (NaCl 150 mM, CaCl2 1 mM, MgCl2 1 mM, NaN3 0.1%, NaF 10 mM, Triton X-100 1% (v/v), ortovanadate 1 mM, aprotinin 2 μg/ml, leupeptin 2 μg/ml, iodoacetamide 10 mM, PMSF 2 mM, and pepstatin 20 μM). The appropriate volumes of 4xSDS-sample buffer and 2-mercaptoethanol 5% (v/v) were then added. Cell lysates were briefly sonicated, warmed at 95°C for 5 min, and cleared by centrifugation at 14.000-g in a microfuge for 15 min at 4°C. Supernatants were collected and proteins were quantified by RC DC protein assay. Equal amounts of proteins were separated from the different samples by SDS-PAGE, and blotted onto nitrocellulose membranes. Anisomycin treated U937 cells were used as positive control for phospho-p38 MAPK detection. Transfer efficiency was checked with Ponceau staining. The blots were blocked in Tris-buffered saline (TBS), containing BSA 2 % (w/v), probed with specific primary antibodies, washed with PBS-Tween 20, and then incubated with a peroxidase-conjugated secondary antibody. Finally, each membrane was probed to detect β–actin. The final dilutions and incubation times suggested by the manufacturer were used for each antibody. Immunodetection was performed using the ECL reagents and Hyperfilm-ECL film.

### Reactive oxygen species (ROS) and cytosolic Ca^++^ detection

CDCF-DA is an oxidation sensitive fluorescent probe, which is first deacetylated inside the cells to the nonfluorescent compound 2’,7’-CDCFH and subsequently can be oxidized to the fluorescent compound 2’,7’-CDCF by a variety of peroxides. For the detection of intracellular Ca^++^ ions we used the calcium-specific probe FLUO-3-AM. These probes were dissolved in anhydrous DMSO at a concentration of 100 mM for CDCF-DA and 1 mM for FLUO-3-AM.

U937 cells were incubated with CDCF-DA (50 μM) or FLUO-3-AM (10 μM) for 30 min. Care was taken that the final DMSO concentration did not exceed 0.1% (v/v). After loading with the probes U937 cells were pelletted, resuspended in complete medium, 1x10^6^/ml, and pretreated or not with KBR (10 μM) or Nifedipine (10 μM) and treated with ouabain for 30 min. ROS or Ca^++^-derived fluorescent signals were detected by flow cytometry (EPICS XL), with excitation and emission settings at 495 and 525 nm, respectively. Fluorescent cells were analyzed on a log scale (FL1) and recorded as mean fluorescence intensity (MFI) of the whole cell population. A minimum of 10.000 events were examined for each sample.

### Statististal analysis

Results are expressed as the means±standard deviation (SD) of repeated experiments, as indicated in the Figure legends. Statistical differences were evaluated using paired 2-tailed Student’s *t* test. Differences were considered statistically significant for values of *P*≤0.05.

## Results

### Effects of low and high doses of ouabain on U937 cells viability

**Figure 1:**
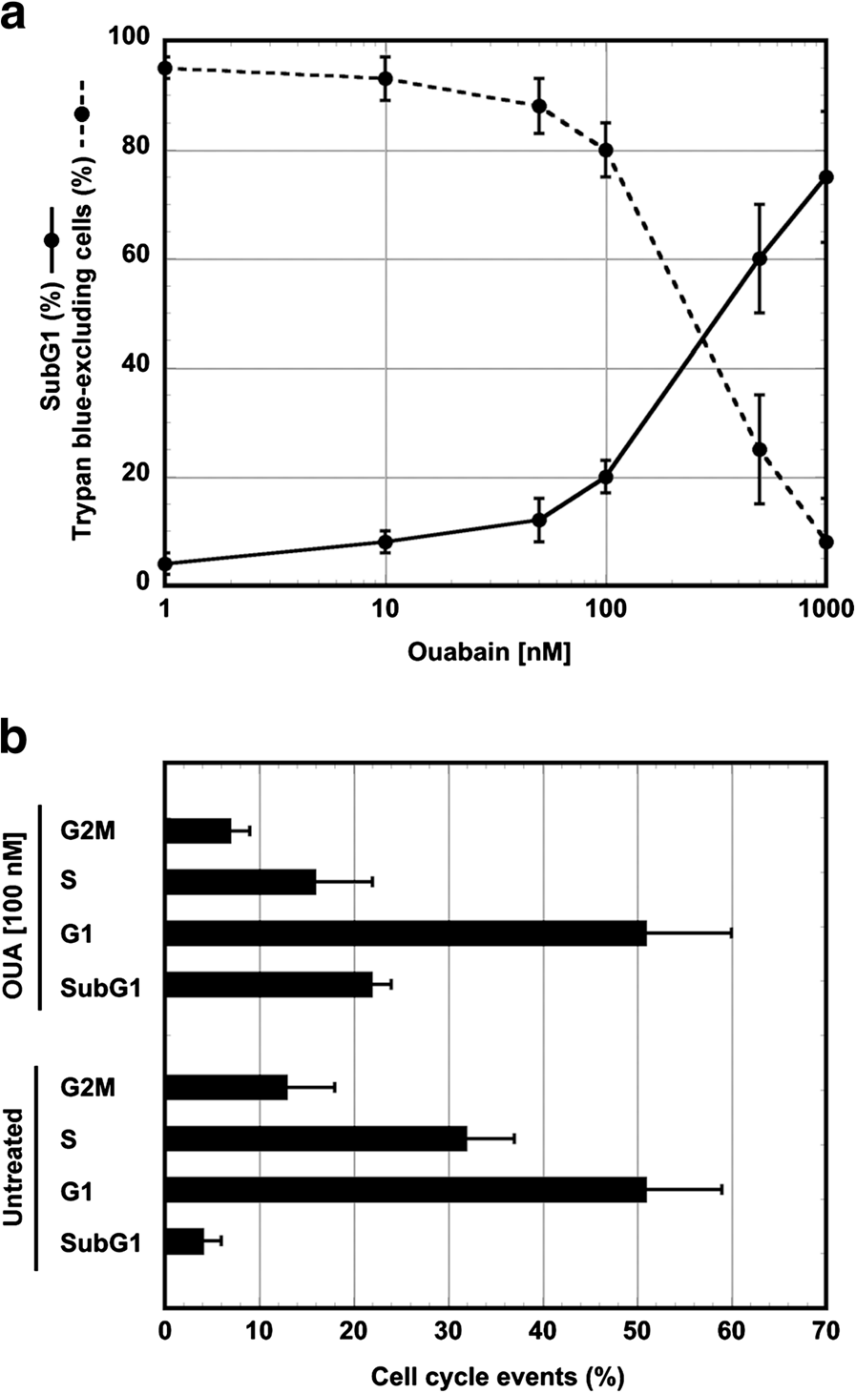
**Cell survival depends on the dose of ouabain.** (**a**) U937 cells were exposed or not to different concentrations of OUA for 24 h. Cells were counted in a hemocytometer as excluding and not excluding trypan blue. Viability was obtained by calculating live cells (trypan blue-excluding) as percentage of all counted cells. A portion of cells were fixed and stained with propidium iodide; subG1 events in the cell cycle were evaluated under cytofluorimetry. The reported values represent the means and the error bars the S.D. of the percentage of live cells (trypan blue-excluding) or subG1 events of six independent experiments. Assessment of cell survival was investigated and statistically significant differences (*P*<0.05) were found between the data obtained using ouabain 100 nM and the two highest concentrations of the drug. (**b**) illustrates the percentage of events in the cell cycle phases of cells untreated or treated with ouabain 100 nM for 24 h. Values are means±S.D. of six experiments.

OUA causes cell death in a dose dependent manner: 24 h treatment with high concentrations of this drug (≥500 nM) resulted cytotoxic for a large proportion of U937 cells, while lower concentrations were less effective, suggesting the activation of a survival pathway (Figures 1a). In particular, OUA 100 nM caused a slight decrease in trypan blue-excluding cells (80±5%) in comparison with untreated cultures (95±2%), in addition to the appearance of 20±3% of subG1 events. SubG1 events were studied by cytofluorimetry of cell cycle phases of cells fixed and stained with propidium iodide: hypodiploid DNA events are easily discernable from the narrow peak of cells with diploid DNA content, and are considered to be indicative of apoptotic nuclei [23,24]. Furthermore, analysis of events in the different cell cycle phases showed that OUA 100 nM caused a decrease in S and G2M phases, while the percentage of G1 events did not change (Figure 1b). Cell counts indicated that at this concentration OUA did not allow cell growth (not shown).

These results suggest that OUA ≥500 nM causes U937 cell death, while OUA 100 nM does not allow cell growth and causes activation of a survival pathway in most U937 cells, increasing the time spent in the G1 cell cycle phase.

### Ouabain causes ROS generation and Ca^++^ elevation

**Figure 2:**
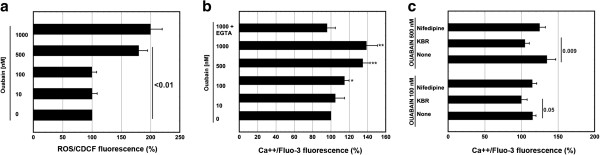
**Ouabain increases the intracellular levels of ROS and Ca**^**++**^**.** (**a**) ROS/CDCF fluorescence as a function of OUA concentration. CDCFH-DA loaded cells were treated with OUA for 30 min. The data are the means ± S.D. of three independent experiments. Statistical analysis by Student’s *t* test is shown. (**b**) Ca^++^/FLUO-3 fluorescence depends on the concentration of OUA and on the cellular entry of the ion. FLUO-3-AM loaded cells were treated with OUA at for 30 min. One cell sample was treated with OUA (1 μM) at the presence of EGTA (2 μM) to discriminate between Ca^++^ entry and Ca^++^ mobilization. The data are the means ± S.D. of five independent experiments. (*, *P* <0.05; **, *P* <0.005 in comparison with untreated cells). (**c**) Intracellular Ca^++^ increase depends on the Na^+^/Ca^++^-exchanger active in the Ca^++^ influx mode. FLUO-3-AM loaded cells were either left untreated or treated with KBR (10 μM) to inhibit NCX or with Nifedipine (10 μM) for 30 min and then with OUA at the indicated concentrations for 30 min. The data are the means ± S.D. of four independent experiments. Statistical analysis by Student’s *t* test is shown. In all experiments the fluorescent signal of **≥**10.000 events was evaluted under cytofluorimetry on a log scale (FL1) and recorded as MFI of the whole cell population. The results are expressed according to the formula (MFI in OUA treated cells)/(MFI in untreated cells) x 100.

Ouabain has been shown to induce ROS generation [12,27] in various cell systems. In comparison with untreated cells we observed a pronounced increase (100±20%) of CDCF fluorescence when U937 cells were treated with ouabain 1 μM and no increase when the concentration of ouabain was ≤500 nM (Figure 2a). Also Ca^++^ elevation has been shown to be caused by cardiac glycosides [4-9,28,29]. We made a similar observation using U937 cells loaded with FLUO-3 and detecting the fluorescence by cytofluorimetry. As shown in Figure 2b, ouabain 1 μM or 100 nM imposed an increase of fluorescence, respectively, of about 39±12% and 15±5% in comparison with untreated cells. Both these data were significant in comparison with those obtained in untreated cells (**, *P*<0.005; *, P<0.05). The increased levels of Ca^++^ were not observed in the presence of EGTA 2 mM in the medium (Figure 2b), indicating the cellular entry of the ion and not its mobilization from internal stores.

NCX is one of the main pathways for intracellular Ca^++^ clearance [9]. However, the inhibition of the Na^+^/K^+^ ATPase by cardiac glycosides, causing the inversion of the Na^+^/K^+^ gradient, leads to impairment of the NCX activity and as a consequence to accumulation of Ca^++^[4-9]. We set out to investigate if NCX was involved in the observed increase of cytoplasmic Ca^++^ following OUA treatment of U937 cells. At this end we used KB-R7943 (KBR) which blocks the Ca^++^ influx mode of NCX rather than the Ca^++^ efflux mode [30,31]. This inhibitor (10 μM) prevented completely the increase of [Ca^++^_i_ caused by OUA (Figure 2c), while the L-type Ca^++^ channel blocker nifedipine (Nif) (10 μM) was ineffective (Figure 2c).

These results were obtained with ouabain either 500 nM or 100 μM, suggesting that also at low concentration OUA impairs NCX, with the result of Ca^++^ entry in the cells.

### NCX promotes cell survival

**Figure 3:**
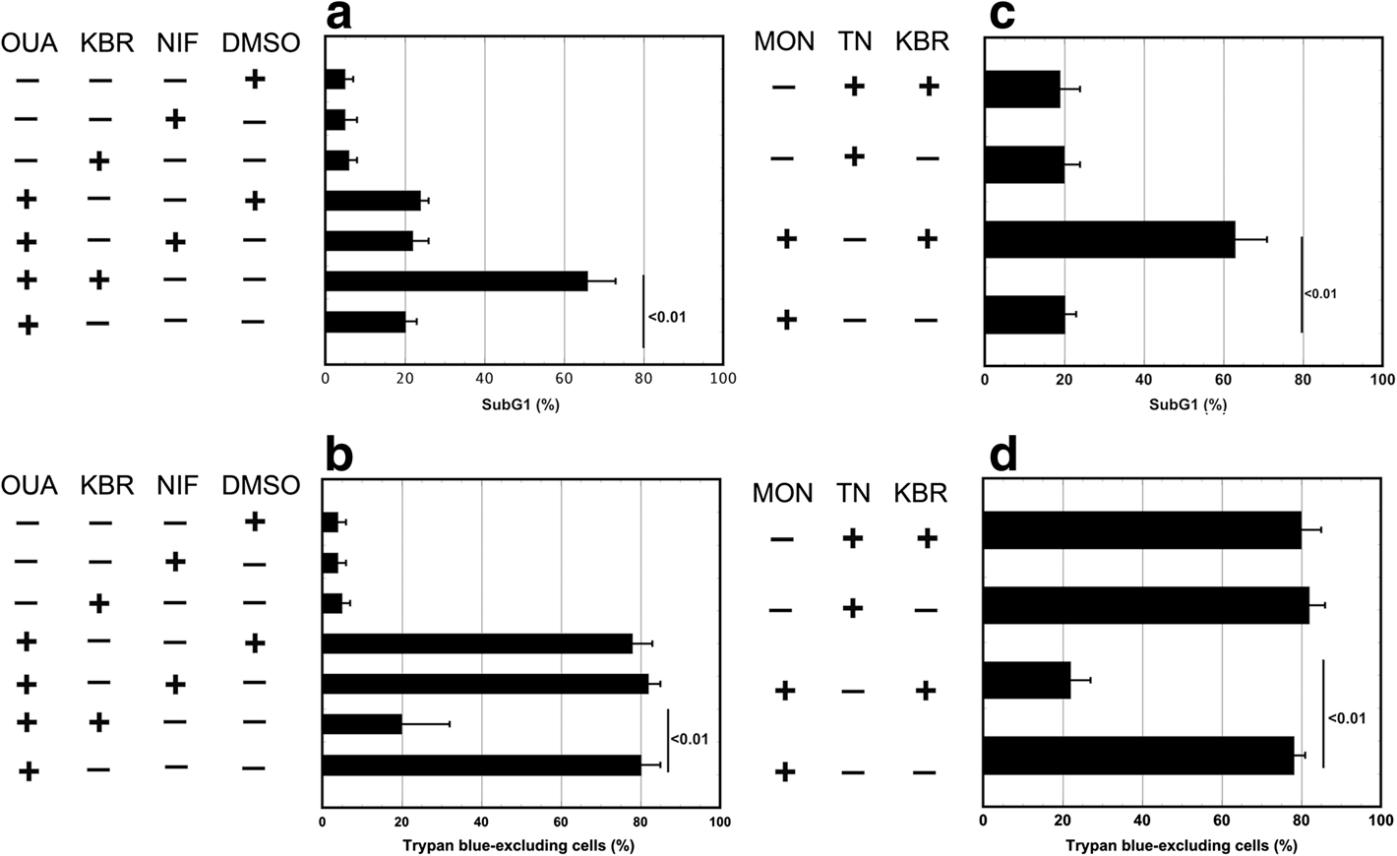
**Survival of U937 cells treated with OUA depends on the activity of NCX.** U937 cells were exposed or not to KBR (10 μM) or to Nifedipine (10 μM) or to DMSO for 30 min and then to OUA 100 nM or again to DMSO for 24 h. (**a**) Cells were fixed and stained with propidium iodide; subG1 events in the cell cycle were evaluated under cytofluorimetry. (**b**) a portion of unfixed cells cells were counted in a hemocytometer as excluding and not excluding trypan blue. Viability was obtained by calculating live (trypan blue-excluding) cells as a percentage of all counted cells. The reported values represent the means and the error bars the S.D. of the percentage of live cells (trypan blue-excluding) or subG1 events of four independent experiments. Assessment of cell survival was investigated and statistically significant differences (*P*<0.01) were found between the data obtained in OUA and in (KBR + OUA) treated cells. (**c**, **d**) U937 cells were pretreated with KBR (10 μM) for 30 min and then exposed to Monensin (3 μM) or Tunicamycin (1 μM) for 24 h. The reported values represent the means and the error bars the SD of the percentage of live cells (trypan blue-excluding) or of subG1 events of four independent experiments. Assessment of cell survival was investigated and statistically significant differences (P<0.01) were found between the data obtained in MON and in (KBR + MON) treated cells.

Cell death was evaluated by detection of trypan blue-excluding cells and of subG1 events in U937 cells pretreated with KBR (10 μM) and then with OUA for 24 h. In particular, NCX inhibition by KBR of U937 cells exposed to OUA 100 nM caused a pronounced increase of cell death (66±7% of subG1 events and 20±15% of trypan blue-excluding cells) in comparison with cells treated only with OUA (20±3% of subG1 events and 80±5% of trypan blue-excluding cells) (Figure 3a,b). Nifedipine (10 μM) did not modify these parameters in comparison with OUA treated cells. Under the same conditions, neither the inhibitors nor DMSO affected cell viability (Figure 3a,b). Monensin (Mon) is a Na^+^ ionophore which causes the entry of Ca^++^ through NCX (L.D.R. unpublished results) [32]. We selected the concentration 5 μM of this drug because it activates a survival pathway in U937 cells resulting in 20±3% of subG1 events and 78±3% of trypan blue-excluding cells (L.D.R. unpublished results). Also in this case the inhibition of NCX by KBR brought upon a pronounced increase of U937 cell death (63±8% of subG1 events and 22±5% of trypan blue-excluding cells) (Figure 3c,d). Tunicamycin (TN) is an ER stressor, which does not impair NCX. At the concentration 1 μM it activates a survival pathway in U937 cells [33], which was not affected by KBR (Figure 3c,d).

Hence, such results allow us to conclude that NCX plays an important role in the pro-survival pathway induced by OUA or monensin.

### Ouabain induces activation of p38 MAPK which plays a pro-survival role

**Figure 4:**
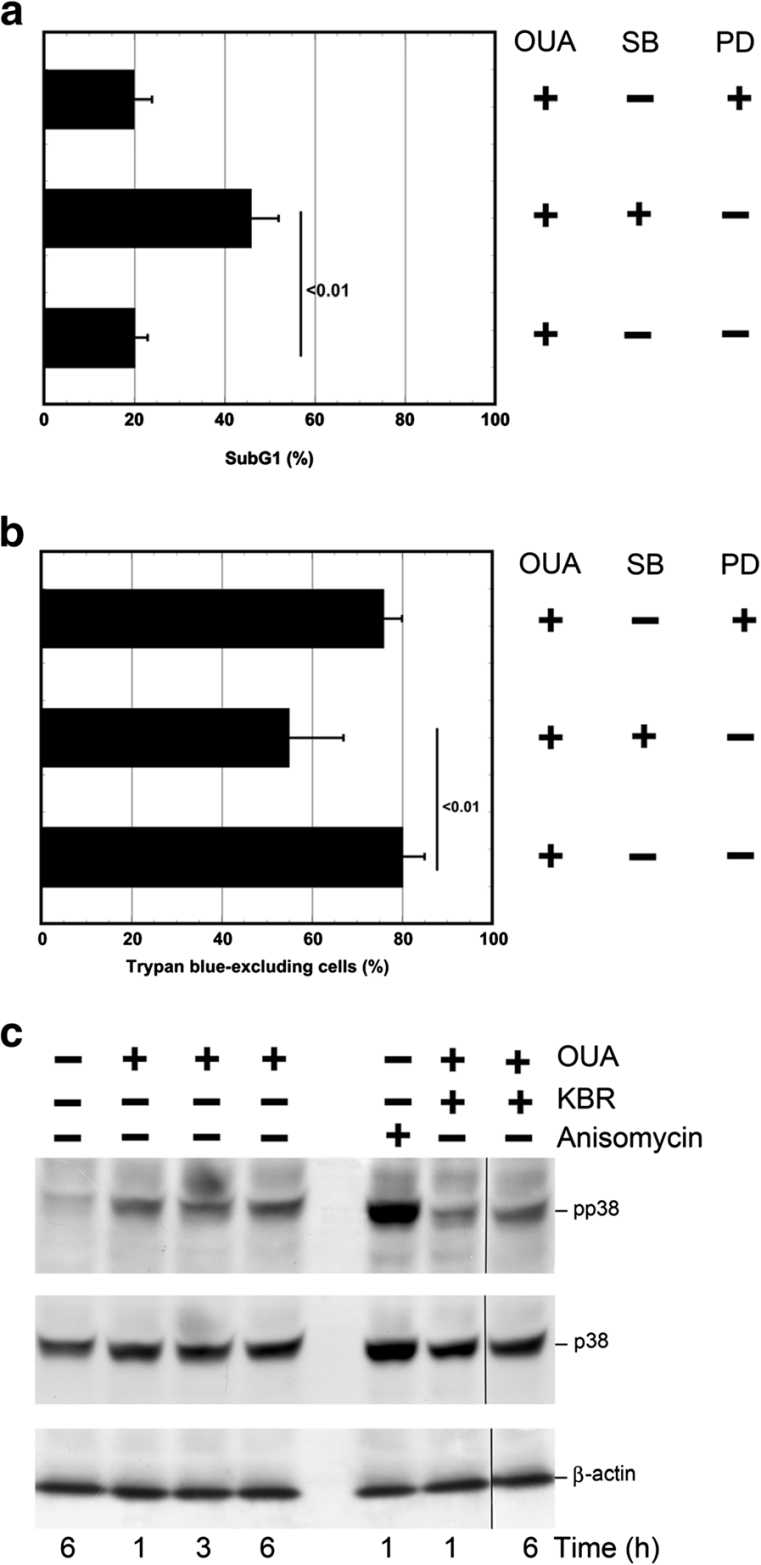
**p38 MAPK is activated and promotes survival in U937 cells.** (**a**, **b**) U937 cells were pretreated with SB203580 (10 μM), inhibitor of p38 MAPK or with PD98059 (10 μM), inhibitor of ERK MAPK for 30 min and then exposed or not to OUA (100 nM) for 24h. (**a**) U937 cells were fixed and stained with propidium iodide; subG1 events in the cell cycle were evaluated under cytofluorimetry. (**b**) A portion of unfixed cells were counted in a hemocytometer as excluding and not excluding trypan blue. Viability was obtained by calculating live (trypan blue-excluding) cells as a percentage of all counted cells. The reported values represent the means and the error bars the SD of the percentage of live cells (trypan blue-excluding) or subG1 events of five independent experiments. Assessment of cell survival was investigated and statistically significant differences (*P*<0.01) were found between the data obtained using OUA and (SB+OUA). **c**) Western blot analysis of activated p38 in the lysates of U937 cells either pretreated or not with KBR (10 μM) and then exposed or not to ouabain 100 nM for the time indicated. Blotted proteins were probed with anti-phospho-p38 and then with anti-p38 antibodies, each followed by peroxidase-conjugated secondary antibody. Anysomicin treated cells were used as positive control for the detection of pp38. The level of β-actin is shown at the bottom as a loading control. One representative experiment of three independent experiments is shown.

MAPK are central mediators of cellular survival and death pathways [33-35]. p38 MAPK can be activated by OUA [36], and by monensin (L.D.R. unpublished results). To investigate the involvement of this MAPK in the above described survival pathway activated by OUA 100 nM, we pretreated U937 cells with SB203580 (SB) 10 μM affecting specifically p38 [37], and then analyzed cell viability. SB203580 pretreatment caused a significant increase of cell death (46±6% of subG1 events and 60±8% of trypan blue excluding cells) in comparison with cells treated only with OUA 100 nM, while pretreatment with the ERK inhibitor PD98059 (PD) 10 μM did not affect cell viability (Figure 4a,b). Under the same conditions, the inhibitors did not affect cell viability (not shown).

To confirm MAPK involvement in the survival pathway activated by the glycoside (100 nM), we performed time-kinetics studies in which phosphorylated p38 and then total p38 were analyzed by western blot with specific antibodies. A faint band of 38 kDa of phospho-p38 proteins was detected in the lysate of untreated U937 cells (Figure 4c), which increased after 1 h, and was still present after 3 and 6 h. When probed with antibodies against total p38, the 38 kDa band showed no change at the investigated time points of OUA treatment, in comparison with that observed in the lysate of untreated cells (Figure 4c). Thus, OUA 100 nM activates p38 MAPK in U937 cells.

Then, we investigated the involvement of NCX in the phosphorylation of p38. However, we did not detect a difference in the band of phospho-p38 in the lysate of cells pretreated with KBR and then with OUA, in comparison with the band observed in the lysate of OUA treated cells (Figure 4c).

Thus, these results suggest that, although p38 plays a pro-survival role in OUA treated cells, its activation is NCX independent.

## Discussion

The first aim of our investigation was to evaluate if OUA is cytotoxic for U937 cells and we detected that at concentrations ≥500 nM it causes ROS generation and a large increase of [Ca^++^]_i_ followed by cell death. We did not explore the link between ROS generation, Ca^++^ increase and cell demise, as it is not surprising that this intracellular milieu can lead to cell death. We were surprised by the survival pathway sparked by lower doses of OUA in which a modest rise of Ca^++^ seems to play an important role. Indeed, U937 cells exposed to ouabain 100 nM were growth arrested in G1 cell cycle phase and escaped from death by activation of a survival pathway, in which were involved the Na^+^/Ca^++^-exchanger active in the Ca^++^ influx mode and p38 MAPK.

It is widely accepted that partial inhibition of the cardiac myocyte Na^+^/K^+^-ATPase by cardiac glycosides causes a modest increase of [Na^+^_i_, which in turn affects the plasma membrane Na^+^/Ca^++^-exchanger, leading to a significant increase of [Ca^++^_i_ and in the force of contraction [4-9]. In the present investigation we show that in U937 cells OUA leads to a rise of [Ca^++^_i_ through NCX active in the Ca^++^ influx mode because this event could be prevented by KBR, an inhibitor known to affect only this type of NCX activity [30,31]. Moreover, OUA became largely cytotoxic after NCX inhibition and not after block of L-type Ca^++^ channel by nifedipine. These conclusions were confirmed treating the cells with the Na^+^ ionophore monensin which, similarly to OUA, causes an increase of [Ca^++^_i_ through NCX active in the Ca^++^ influx mode. Finally, the endoplasmic reticulum stressor tunicamycin, not affecting NCX, proved to be a good control because it induced cell death in a low proportion of cells, not increased by KBR.

MAPK are central mediators of cellular survival and death pathways [33-36]. To investigate their involvement in the survival pathway activated by OUA, we pretreated the cells with inhibitors at concentrations affecting specifically one MAPK and then analyzed cell viability. These experiments indicated that p38 plays a pro-survival role in OUA treated cells. It has been reported that a phospholipase A2 (PLA2), modified in order to loose the catalytic activity, can induce apoptosis in U937 cells through a catalytic activity-independent pathway, in which plays a relevant role the activation of p38 MAPK dependent on the elevation of intracellular Ca^++^ levels [38]. However, those results are different from ours, as nifedipine abrogated Ca^++^ increase and rescued viability of U937 cells, while we observed that nifedipine does not abrogate Ca^++^ rise and does not modify cell viability, while KBR prevents Ca^++^ rise and increases cell death. Thus, we would roule out the involvement of a PLA2 catalytic activity-independent pathway in the activation of p38 by ouabain, even if we did not detect the link between NCX and p38 phosphorylation.

At the present we can affirm that OUA activates a pro-survival pathway in which NCX active in the Ca^++^ influx mode is necessary, but we cannot conclude that is essential the [Ca^++^]_i_ rise. We can speculate that Ca^++^ influx through NCX may function as a second messanger responsible of a molecular pathway leading to cell survival.

This work shows that the cardiac glycoside OUA is cytotoxic also for the lymphoma derived cell line U937 and suggests to consider that at lower concentration this drug activates a survival pathway in which NCX and p38 MAPK can represent potential targets of combined therapy.

## Abbreviations

ECL: Enhanced chemiluminescence; FCS: Fetal calf serum; HRP: Horseradish peroxidase; KBR: KB-R7943; MAPK: Mitogen-activated protein kinase; MFI: Mean fluorescence intensity; Mon: Monensin; NCX: Na^+^/Ca^++^-exchanger; Nif: Nifedipine; OUA: Ouabain; PBS: Phosphate buffered saline; PD: PD98059; PI: Propidium iodide; ROS: Radical Oxygen Species; SB: SB203580; SDS-PAGE: Sodium-dodecyl-sulphate-polyacrylamide gel electrophoresis; TN: Tunicamycin.

## Competing interests

The authors declare that they have no competing interests.

## Authors’ contributions

CF, RM, BL, PR, LDR performed most of the experiments. CF, RM and LDR contributed to the conception and design of the experiments, to the analysis and interpretation of the data. LDR wrote the manuscript. All authors read and approved the final manuscript.
